# A new approach to the lower cervical-thoracic spine with dislocation of the sterno-clavicular joint: FAMA (Fast Anterior Medium Approach)

**DOI:** 10.1007/s00264-026-06750-1

**Published:** 2026-03-19

**Authors:** Fabrizio Cuzzocrea, Gianluigi Pasta, Alessandra Monzio Compagnoni, Micaela Berni, Salvatore Annunziata, Matteo Ghiara, Mario Mosconi, Gino Volpato

**Affiliations:** 1 Orthopedics and Traumatology Clinic, IRCCS Policlinico San Matteo Foundation, Pavia, Italy; 2 Department of Clinical, Surgical, Diagnostic and Pediatric Sciences, University of Pavia, Pavia, Italy; 3 Department of Medicine and Surgery, IRCCS Policlinico San Matteo Foundation, Pavia, Italy

**Keywords:** Lower cervical-thoracic spine, Sterno-clavicular joint dislocation, Fast Anterior Medium Approach, FAMA, Surgical technique

## Abstract

**Purpose:**

To evaluate the Fast Anterior Medium Approach (FAMA) as an alternative to traditional anterior cervico-thoracic approaches, enhancing access to the C7-T1-T2 and T2-T4 junctions while minimizing postoperative morbidity. The cervico-thoracic junction is one of the most challenging regions to access surgically due to its deep location and proximity to critical neurovascular structures. Conventional approaches, including postero-lateral thoracotomy and transmanubrial techniques, are associated with high morbidity. The FAMA technique was designed to provide enhanced exposure while reducing surgical trauma.

**Methods:**

A cadaveric study was performed to understand how FAMA approach could find application in spine surgery in order to obtain wider access to the cervico-thoracic spine with lower post-operative morbidity compared to the surgical procedure with sternotomy. This approach involves controlled dislocation of the sterno-clavicular joint to extend anterior access without requiring sternotomy.

**Results:**

The approach allowed excellent exposure of the thoracic apex, enabling safe spinal stabilization procedures with minimal disruption to surrounding structures. No major neurovascular injuries occurred.

**Conclusion:**

The FAMA approach represents a viable alternative to conventional cervico-thoracic surgical techniques, offering improved visualization and accessibility while preserving anatomical integrity. This technique has the potential to reduce morbidity and improve patient recovery. Larger-scale studies are required to validate these findings.

## Introduction

**Fig. 1:**
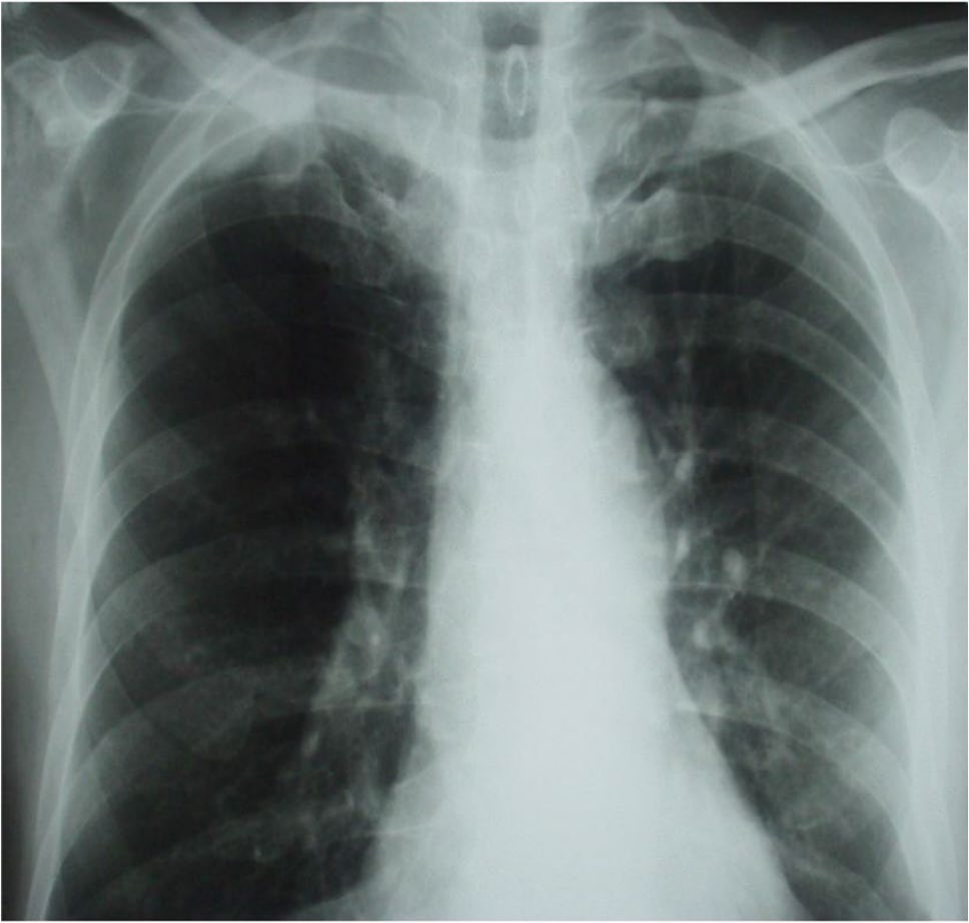
AP radiographs image of the chest

Surgical access to the thoracic apex has long posed a technical challenge due to its deep anatomical location and proximity to critical neurovascular structures. Traditional postero-lateral thoracotomy often requires extensive dissection of the serratus anterior and trapezius muscles, along with scapular hyperextension. Historically, in the treatment of pulmonary tuberculosis, a ceiling hook was used to suspend the scapula, enhancing exposure (Fig. 1) [1].

A similar issue arises in the resection of superior pulmonary sulcus tumors (Pancoast tumours), which also demand precise dissection in this anatomically restricted zone [2]. Given the limitations of traditional thoracotomy, various anterior approaches have been proposed.

Although available techniques [3, 4] provide better exposure, they are associated with specific drawbacks. Clavicular resection may lead to suboptimal cosmetic outcomes, while manubrial access increases the invasiveness and morbidity of the procedure.

Moreover, anterior approaches to the cervico-thoracic spine are highly dependent on the involved spinal level. The C3–C6 levels are accessible through a low cervicotomy, whereas access to C7–T1 requires skeletonization of the manubrium. In roughly 25% of cases, surgical exposure of T2–T4 necessitates partial or total sternotomy [5].

To address these limitations, a new surgical approach was originally concieved by Prof. Volpato and used on 12 patients to obtain the resection of a Pancoast tumor without performing sternotomy but only dislocating sternoclavicular joint and then reducing it. This reproducible and minimally invasive technique—FAMA—designed to provide fast (Fast), anterior (Anterior), and mid-thoracic (Medium) access for surgical procedures targeting the C7 to T4 vertebral levels that avoids sternotomy and clavicular resection. Instead, it uses selective disarticulation and superior dislocation of the sterno-clavicular joint to allow wide access to the thoracic apex, facilitating the dissection of retroclavicular and subclavicular structures and direct access to the upper pulmonary lobe.

We performed a study on cadavers in order to understand how FAMA approach could find application in spine surgery in order to obtain wider access to the cervico-thoracic spine with lower post-operative morbidity compared to the surgical procedure with sternotomy.

In this manuscript, we describe the surgical technique and a cadaveric study reporting preliminary results on its application in spine surgery in order to obtain wider access to the cervico-thoracic spine with lower post-operative morbidity compared to the surgical procedure with sternotomy.

## Materials and methods

### Surgical technique

**Fig. 2:**
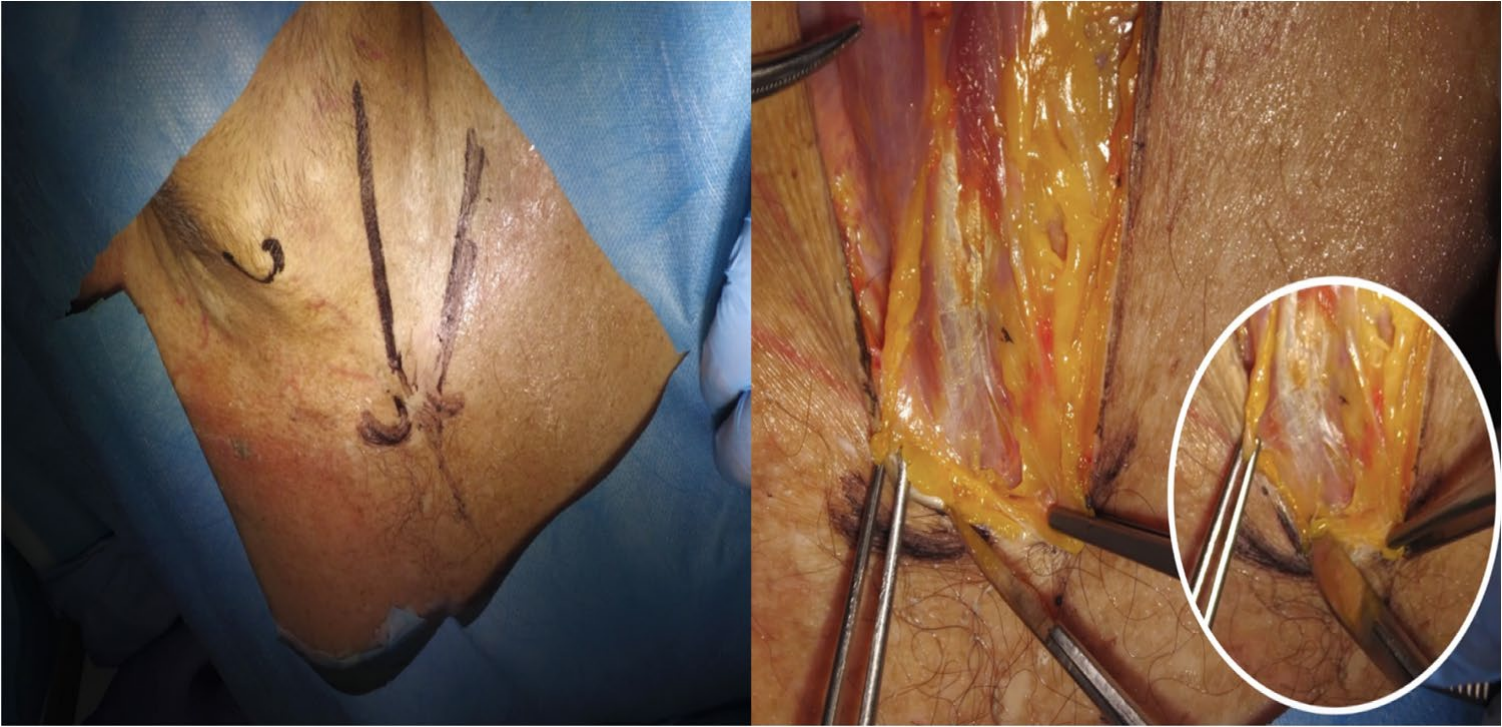
**A**) The approach is a sort of L-shaped skin incision, with the vertical branch along the medial edge of the SCM muscle, and the horizontal branch on the medial third of the clavicle. **B**) The sternum-claveare is exposed by identifying the capsule

The patient was placed in a supine position with a small support under the shoulders and the head extended and rotated contralaterally. After standard sterile preparation, an L-shaped skin incision was performed, consisting of a longitudinal component along the medial border of the sternocleidomastoid muscle and a transverse limb across the medial third of the clavicle (Fig. 2A) [6].

The platysma was incised, and the superficial cervical fascia was dissected. The sternocleidomastoid and strap muscles were retracted laterally, exposing the sterno-clavicular joint. The clavicular periosteum was carefully elevated to protect the subclavian vessels and brachiocephalic vein [5]. The costoclavicular ligament was then sectioned extracapsularly, allowing for controlled mobilization of the clavicle [7].

**Fig. 3:**
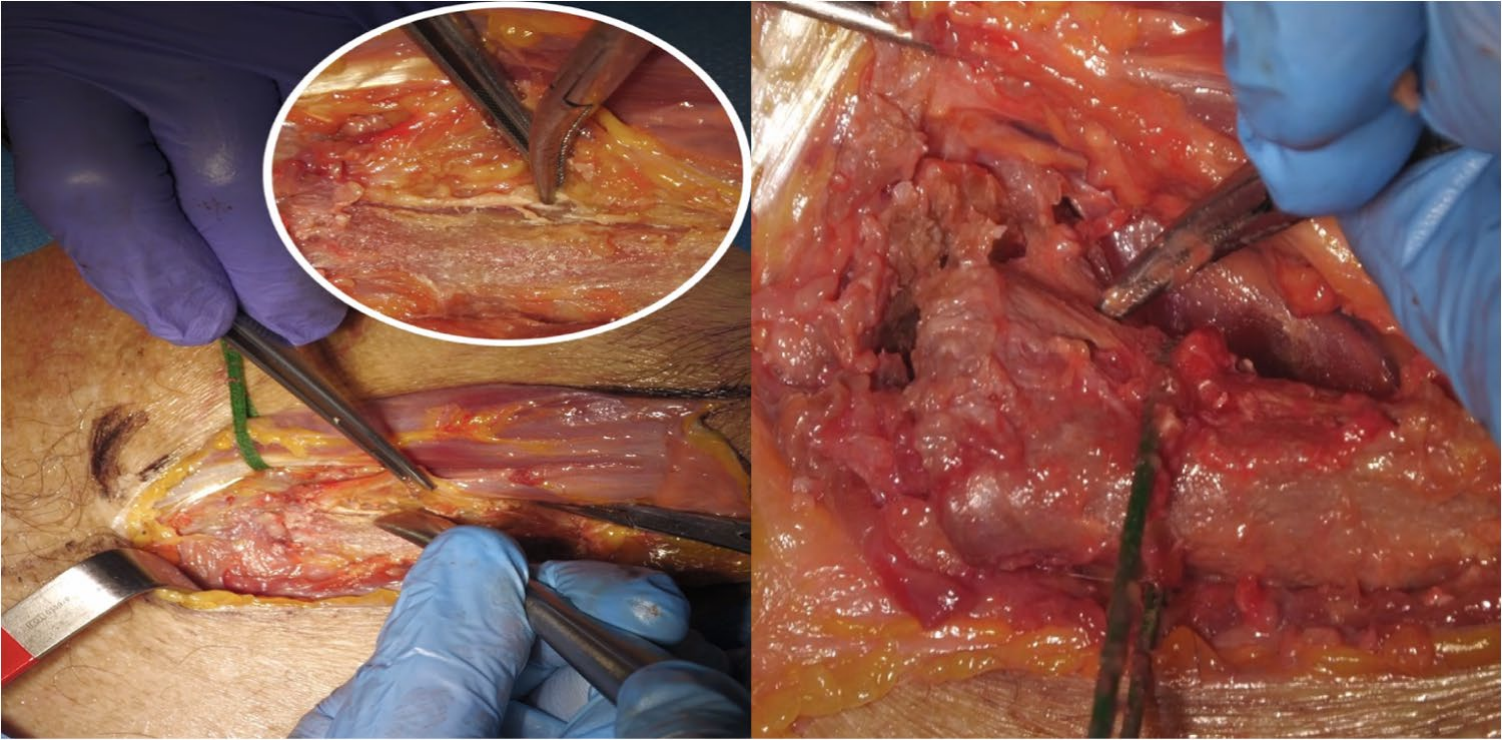
**A**) Exposing the clavicle trying to keep the periosteum intact. **B**) Incision of the articular capsule of the sternum-claveare freeing it back with a curved Klemmer until mobilizing it

**Fig. 4:**
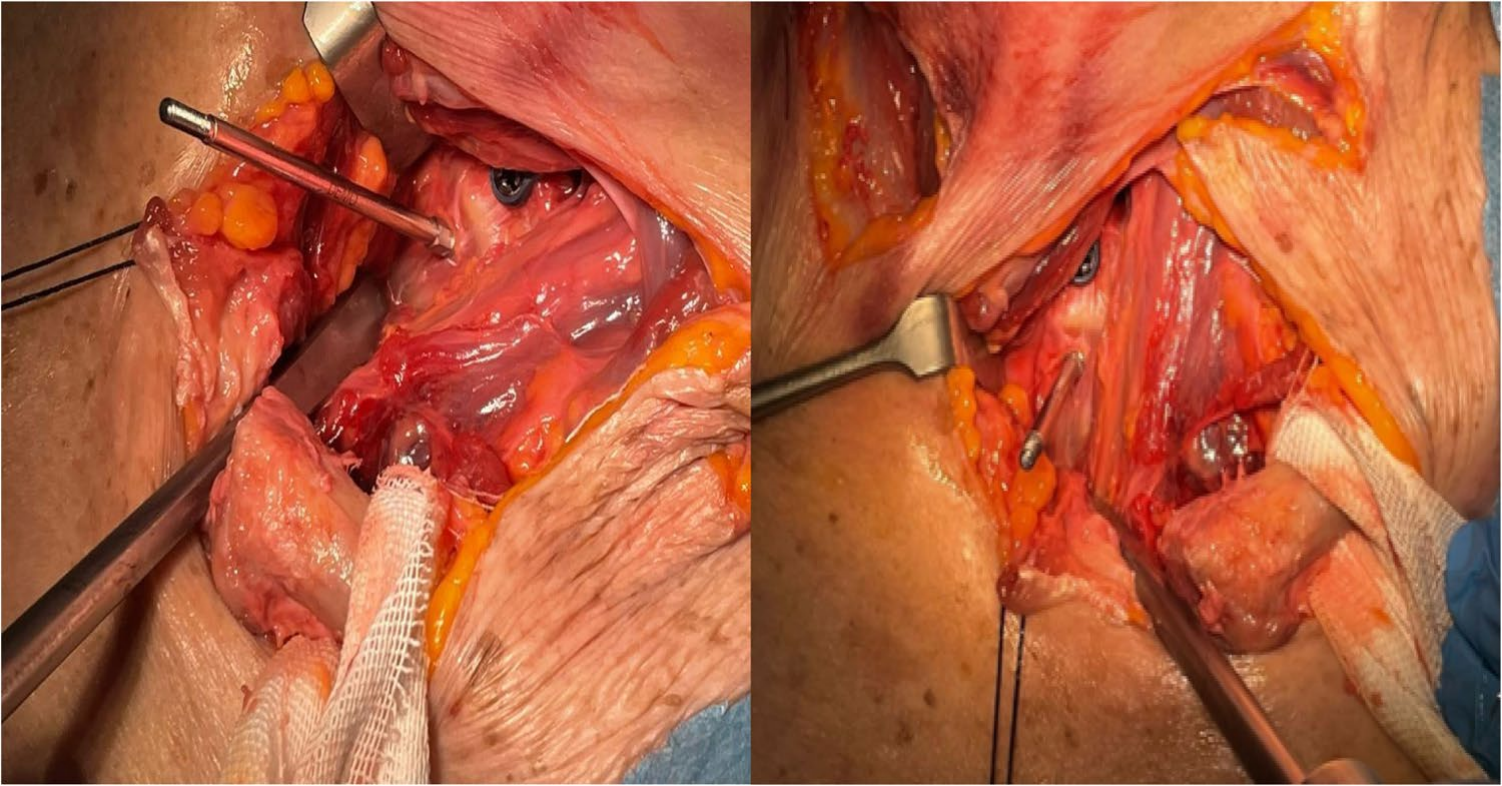
Mobilization of the clavicle after hanging it with a ligature (wrapper) and exposure of the anterior cervical spine. A pin was used as a repere that was placed through the cervical access: after dislocation of the sternum-claveare repositions the metal repere to see the lower space. By placing medially on the clavicle close to the sternum, it is possible to gain the midline

The joint capsule was incised, and the medial clavicle was gently dislocated superiorly and posteriorly, improving access to the retroclavicular and upper mediastinal space (Fig. 2B) [4]. With the clavicle retracted, a wide corridor to the thoracic apex was obtained, enabling exposure of the C7 to T4 vertebral bodies (Figs. 3 and 4).

**Fig. 5:**
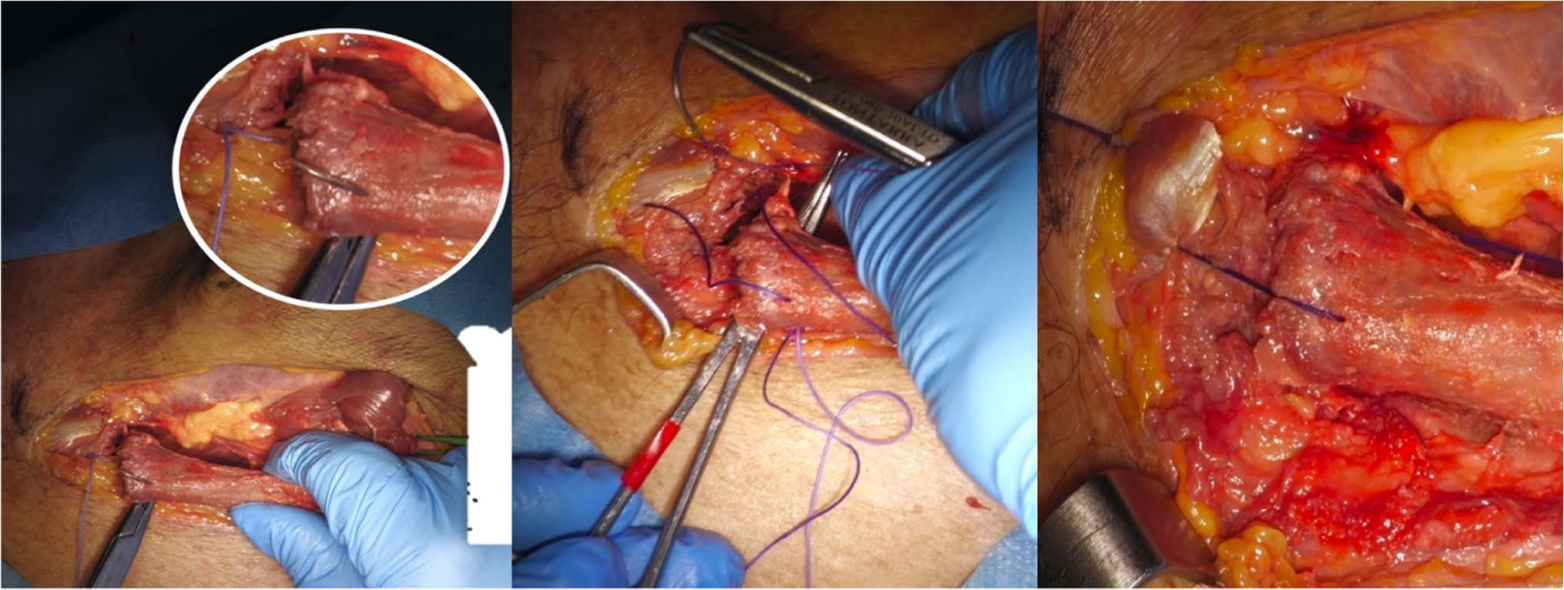
The repositioning of the clavicle that is fixed with trans-bony points at the distal end of the clavicle and the level of the sternal handlebar. Performing a tie and stabilizing the clavicle by Vycril of 2

Following decompression and instrumentation, the clavicle was repositioned. Transosseous sutures (Vycril 2) were used to reattach the joint capsule and restore joint stability (Fig. 5). Layered closure was completed with attention to soft tissue alignment to minimize scarring and preserve aesthetics (Table 1).

**Table 1:** Comparative Table of different approaches

Approach	Exposure	Invasiveness	Cosmetic Impact
Dartevelle	C7–T2	High (clavicle resection)	Moderate
Transmanubrial	C7–T4	High (sternotomy)	Significant
FAMA	C7–T4	Low (joint dislocation)	Minimal

## Results

The approach allowed excellent exposure of the thoracic apex, enabling safe spinal stabilization procedures with minimal disruption to surrounding structures. No major neurovascular injuries occurred.

## Discussion

Anterior approaches to the cervical and cervico-thoracic spine carry known risks, including oesophageal injury (e.g., fistula formation), recurrent or superior laryngeal nerve palsy, vascular injury, and heterotopic ossification of the longitudinal ligaments [8].

Access to the first thoracic vertebrae has historically been considered one of the most complex aspects of anterior spinal surgery.

The Fast Anterior Medium Approach (FAMA) emerges as a strategic modification designed to overcome these limitations. By dislocating the sterno-clavicular joint without resecting the clavicle or opening the sternum, FAMA minimizes surgical trauma and preserves thoracic integrity. This is especially relevant for elderly patients or those with comorbidities, where minimizing invasiveness can have a direct impact on outcomes.

In this study, the FAMA technique proved effective in providing a safe surgical corridor with excellent visualization of the upper thoracic vertebrae. The approach allowed efficient handling of various pathologies—including tumours, degenerative stenosis, and infectious processes—demonstrating versatility in its indications. Notably, the approach avoided the need for manubriotomy or clavicular resection.

The main technical demands of FAMA include the identification and protection of key anatomical structures, such as the brachiocephalic veins, internal thoracic artery, phrenic nerve, and recurrent laryngeal nerve. A right-sided approach is generally preferred to reduce the risk of thoracic duct injury. In our experience, careful blunt dissection and the use of magnification enabled safe passage through these critical zones.

Our findings suggest that FAMA may serve as a valuable alternative for anterior access to the cervico-thoracic junction. Further comparative studies with in vivo larger cohorts—particularly those evaluating long-term biomechanical consequences, postoperative pain, and return to function—are needed to confirm its long-term efficacy and broader applicability.

Moreover, with the growing application of robotic and minimally invasive techniques in spinal surgery, FAMA offers a foundational route that may be adapted to future technologies. Its relatively straightforward learning curve makes it a feasible option for spine surgeons familiar with anterior cervical approaches.

## Data Availability

No datasets were generated or analysed during the current study.
